# Novel expandable short dental implants in situations with reduced vertical bone height—technical note and first results

**DOI:** 10.1186/s40729-017-0107-1

**Published:** 2017-10-30

**Authors:** Waldemar Reich, Ramona Schweyen, Christian Heinzelmann, Jeremias Hey, Bilal Al-Nawas, Alexander Walter Eckert

**Affiliations:** 1 Department of Oral and Plastic Maxillofacial Surgery, Martin Luther University Halle-Wittenberg, Ernst-Grube Str. 40, 06120 Halle (Saale), Germany; 2 University School of Dental Medicine, Department of Prosthetic Dentistry, Martin Luther University Halle-Wittenberg, Magdeburger Straße 16, 06112 Halle (Saale), Germany

**Keywords:** Bone atrophy, Expandable, Macrodesign, Short implant, Implant stability

## Abstract

**Purpose:**

Short implants often have the disadvantage of reduced primary stability. The present study was conducted to evaluate the feasibility and safety of a new expandable short dental implant system intended to increase primary stability.

**Methods:**

As a “proof of concept”, a prospective clinical cohort study was designed to investigate intraoperative handling, primary and secondary implant stability (resonance frequency analysis), crestal bone changes, implant survival and implant success, of an innovative short expandable screw implant. From 2014 until 2015, 9 patients (7–9-mm vertical bone height) with 30 implants (length 5–7 mm, diameter 3.75–4.1 mm) were recruited consecutively.

**Results:**

All 30 implants in the 9 patients (age 44 to 80 years) could be inserted and expanded without intraoperative problems. Over the 3-year follow-up period, the implant success rate was 28/30 (93.3%). The mean implant stability quotients (ISQ) were as follows: primary stability, 69.7 ± 10.3 ISQ units, and secondary stability, 69.8 ± 10.2 ISQ units (*p* = 0.780), both without significant differences between the maxilla and mandible (*p* ≥ 0.780). The mean crestal bone changes after loading were (each measured from the baseline) as follows: in the first year, 1.0 ± 0.9 mm in the maxilla and 0.7 ± 0.4 mm in the mandible, and in the second year, 1.3 ± 0.8 mm and 1.0 ± 0.7 mm, respectively.

**Conclusions:**

Compared to other prospective studies, in this indication, the success rate is acceptable. Implant stability shows high initial and secondary stability values. The system might present an extension of functional rehabilitation to the group of elderly patients with limited vertical bone height. Further long-term investigations should directly compare this compressive implant with standard short implants.

**Electronic supplementary material:**

The online version of this article (10.1186/s40729-017-0107-1) contains supplementary material, which is available to authorized users.

## Introduction

Endosseous implants have been established over several decades. The evaluation of treatment results under biomechanical, physiological, psychological, social and economic aspects has been well documented [1]. Furthermore, patient-based outcomes reveal a predictable gain in oral health-related quality of life [2].

Especially in patients with limited vertical bone height, process of treatment is extensive. Prior to implantation, augmentation procedures are required [3]. Depending on gender, vascularisation and bone mineralisation up to 25% of the primary volume are resorbed due to remodeling of augmented alveolar ridges [4]. Recently, short dental implants have evolved into a promising and reliable treatment option in the orofacial rehabilitation of atrophic mandibles and maxillae, namely as an alternative to vertical ridge augmentation [5–8]. The prognosis of short implants and patient satisfaction is high [9–12].

The definition of short implants in the literature is not uniform. In this present study, we considered short implants with 5–8-mm length [5, 7, 13]. Other authors set the cut-off at 6 mm [8, 9, 11, 14, 15]. According to the recent consensus paper of the 11th European Consensus Conference (EuCC), dental implants are referred to as “short” if their intrabony length measures ≤ 8 mm and considered as “ultra-short” with lengths < 6 mm [16].

Biomechanical studies show that the crestal bone is strained under axial and extra-axial loading [17]. While bone quality, implant design and position, prosthetic devices and material characteristics contribute to the character of stress distribution, the role of implant length seems to be of underpart [17, 18]. Nevertheless, implant length is crucial in D4 bone quality [19], and the crown-to-implant length itself influences stress distribution under *extra-axial* loading in the crestal bone [20] and in the abutment screw [21]. According to Petrie and Williams [22], the influence of increased implant diameter on stress reduction in the crestal bone is more efficient than increased implant length. Möhlhenrich and co-authors [23] confirmed these findings that the diameter of an implant has greater influence on primary stability than implant length. Based on in vitro analysis, they concluded additionally that especially in patients with poor bone quality, a variation of implant dimensions is expected to lead to a significant increase of primary stability. Furthermore, stress distribution on short implants is affected by the bone-to-implant contact ratio [24]. Consequently, several options to increase the implant surface of short implants are elaborated, which consecutively enhance the implant stability: thread number, thread shape, thread depth, implant diameter, implant design and surface topography [25–27].

It is known that achievement of primary stability is one precondition for osseointegration and treatment success. There are few reports of immediate [14] and early (6 weeks) functional loading of short implants [28]. This is related to good bone quality, implant design or implant site preparation (e.g. under-drilling). However, under-drilling of the crestal aspect may lead to decreased bone-to-implant contact [29]. It is desirable to reduce the periimplant stress on the crestal bone while providing sufficient primary stability for all bone densities.

Therefore, optimisation of the macro- and microdesign of short dental implants to improve the success rate and long-term stability is preferable. In fact, elderly patients with general comorbidity should benefit from the overall short treatment time [28, 30]. As previously shown, the oral health-related quality of life is compromised during the healing period after implant insertion [31], especially when augmentation procedures are required [11]. For several reasons, the overall treatment time should be reduced in patients with atrophic alveolar ridges.

The purpose of the present study was to clinically analyse the feasibility and safety of a new short dental implant system with an expandable compressive design in the apical region. We hypothesised that the innovative expandable macrodesign of this implant provides a reliable implant success rate and ensures high implant stability in vivo.

## Material and methods

### Study population and measures

The study was designed as a prospective monocentric longitudinal cohort study according to the STROBE criteria. The participants of this study were recruited at the university hospital of Martin Luther University Halle-Wittenberg, Department of Oral and Plastic Maxillofacial Surgery, implantological consultation from 2014 (June) until 2015 (June). Inclusion and exclusion criteria of adult patients interested in implantological treatment are summarised in Table 1. Written informed consent was obtained from all individual participants included in this study.

**Table 1:** Patient recruitment

Inclusion criteria	Exclusion criteria
1. Adult patients, male and female	1. Comorbidity ASA category ≥ III
2. Partially/totally edentulous patients	2. Pregnancy, bruxism
3. Alveolar process atrophy Cawood et Howell category ≥ IV	3. Smoking ≥ 10 cigarettes/day
4. Minimum vertical bone height of 7–9 mm for placement of short implants (5–7-mm length)	4. Radiotherapy ≥ 50 Gy [72] or 5. Intravenous bisphosphonate therapy [73] with a significant risk of developing osteo(radio)necrosis of the jaw
5. Patients without willingness to accept vertical bone augmentation	6. Psychiatric comorbidity that could influence course of treatment
	7. Untreated or poorly controlled diabetes mellitus
	8. Highly atrophic jaws that require vertical augmentation

As a “proof of concept”, the pilot study was designed to investigate the intraoperative handling and to evaluate the feasibility and safety of a new short implant system. Therefore, sample size calculation was not performed. The primary outcome variable was implant success rate, which was calculated considering known success criteria (implant in function, no sign of infection or pain, no mobility, no radiolucent area around the implant) [32, 33]. The implant survival was calculated according to the Kaplan-Meyer method. Secondary measures were implant stability (initial and secondary) and periimplant crestal bone changes. Implant stability was measured by resonance frequency analysis (RFA; Osstell AB, Göteborg, Sweden).

Primary stability was measured immediately after implant insertion and completed expansion (see below), and secondary stability after the submerged healing period (3 months in the mandible, 6 months in the maxilla; Table 2) during the re-entry operation just before the healing abutments were inserted. Implant stability quotient (ISQ) values were obtained using the Smartpegs (type 17 and 35). According to each measurement, implant stability was classified as low with ISQ values < 60, medium with ISQ values 60–70, and high with ISQ values > 70 [34].

**Table 2:** Surgical treatment protocol

Surgical protocol	Bone quality			
	D1	D2	D3	D4
1. Drilling sequence (splint)	Last drill	Last drill	Second to last drill	Second to last drill
2. Condensing preparation	–	–	(Analogue to last drill)	Analogue to last drill
3. Implant insertion (maximum torque ≤ 40 N cm)				
4. Expansion (maximum torque ≤ 40 N cm)				
5. Evaluation of primary stability by resonance frequency analysis, primary wound closure				
6. Postoperative digital radiogram				
7. Re-entry after a conventional period of submerged healing (mandible 3 months, maxilla 6 months), evaluation of secondary stability by resonance frequency analysis and insertion of healing abutments				
8. Postoperative digital radiogram				

Digital radiograms (orthopantomogram, standard periapical radiograms) were taken prior to surgery, after implant insertion and re-entry, and at yearly follow-up examinations for crestal and periapical bone evaluation (see below).

### Implants

**Fig. 1:**
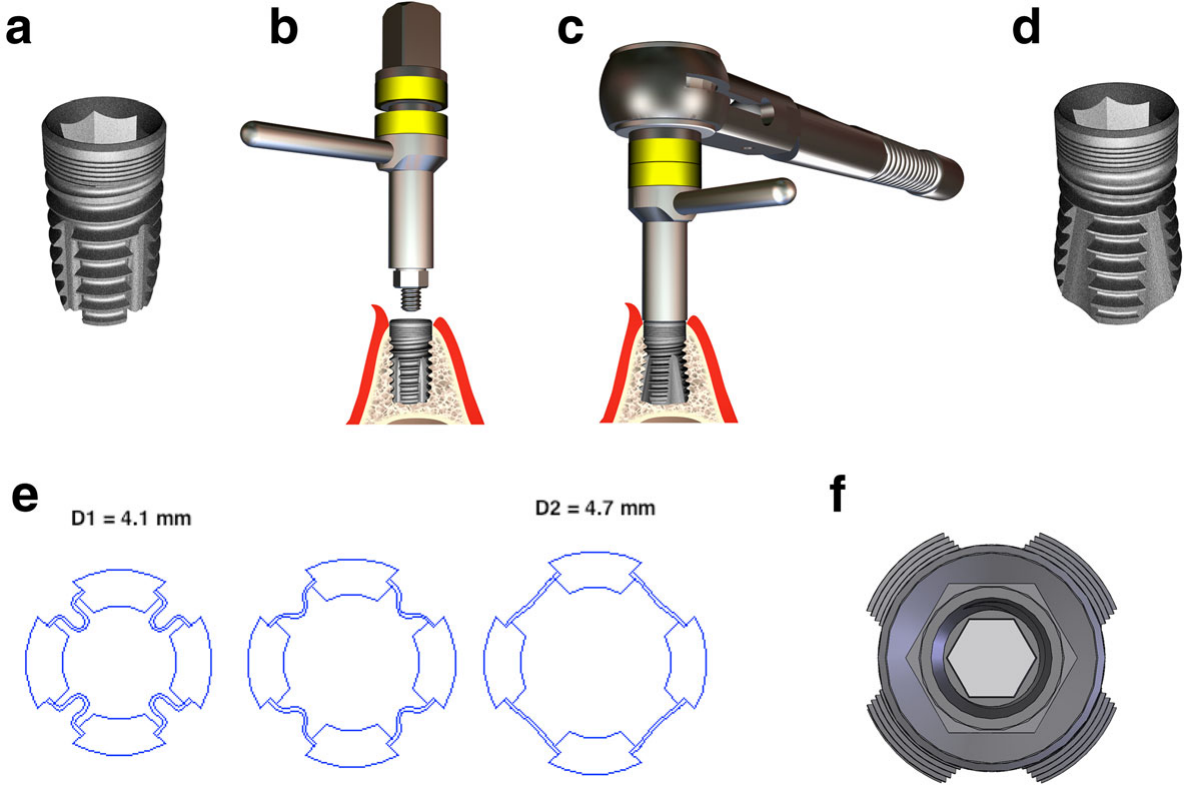
**a** Closed short expandable dental implant (4.1 × 7 mm). The implant-abutment connection is characterised by an internal hexagon for rotation stability, combining the advantages of conical and parallel surfaces to reduce microgaps and micromovement [68]. The microthread concept and platform switching concept are implemented in the implant shoulder to reduce periimplant bone strain [53]. **b** Manual fixation of the expansion tool. Take note of the distance between both *yellow rings*. **c** Completion of the expansion process using the ratchet. Take note of the contact between both *yellow rings*. **d** Opened short expandable dental implant (4.1 × 7 mm). The expanded implant provides an increased bone-to-implant interface (*pyramid shape*) in the apical portion [54]. **e** Cross-section view of the implant apex. The apical expansion process is characterised by the unfolding of four wings, which are connected by four foils. D1: diameter of the closed implant. D2: diameter of the opened implant. **f** *Top view* of the expanded implant. The expanded implant (4.1-mm diameter) displays an apical diameter of 4.7 mm and length of the edge (base) of 4.4 mm

In this study, a short expandable titanium screw implant (PYRAMIDION dental implant, DenTack Implants Ltd., Kfar-Saba, Israel) was used, which leads to dynamic condensing of the apical bone. The implants had the following dimensions and special characteristics: 5, 6 and 7 mm in length, 3.75 and 4.1 mm in diameter and an internal (7-mm length) or external (5- and 6-mm length) hexagon platform. The apical expansion is performed after implant insertion using a special expansion tool and a ratchet torque, resulting in a pyramid shape (Fig. 1a–f). The implant expansion process using the expansion tool is visualised in the movie clip (Additional file 1).Additional file 1:Simulation of the expansion process. At the end of the expansion process, a minimal snap back is realised. (MP4 9407 kb)

### Surgical and prosthetic protocol

Planning of the implantological treatment followed usual clinical and radiological examination and, concerning the position and number of implants, the recommended categories from the German consensus conference [35]. The drilling sequence, condensing preparation (where necessary) and manual implant insertion as well as expansion are described in detail in Table 2. Participants were instructed not to wear their denture 1 week after surgery. Afterwards, the conventional dentures were relined with soft material (Visco-gel, Dentsply, Salzburg, Austria). In this study, conventional periods of submerged healing were chosen: 3 months in the mandible and 6 months in the maxilla. During the re-entry surgery, a minimum of 2-mm keratinised periimplant soft tissue mucosa was considered.

All prosthetic treatments were provided at the University School of Dental Medicine, Department of Prosthetic Dentistry. At the earliest, 2 weeks after surgical re-entry, prosthetic treatment was started. All treatment steps were performed as described in detail in Table 3. The abutment screws were fixed with a torque of 15 N cm. Wherever possible, adjacent implants were primarily splinted (crowns, bar) and *extra-axial* loading during dynamic occlusion was avoided. In other cases, eccentric group guidance was achieved. To reduce overloading in the periimplant bone and implant-abutment connection, the occlusal surface was designed smaller [20, 21, 30, 36]. Patients were instructed about optimal oral hygiene, and the use of a dental water jet was recommended.

**Table 3:** Prosthetic treatment protocol

Type of prosthetic treatment		Session	Procedure
Fixed denture (bridge)		1	Open impression
		2	Abutment check, set-up • Titanium abutments • Non-precious metal framework, completely lined • Neighbouring crowns interlocked
		3	Check and insertion of the suprastructure • Cementation (ImProv™, Dentegris, Duisburg, Germany)
Combined fixed-removable denture	Telescope	1	Open impression (implants and stumps)
		2	Jaw relation (wax splint)
		3	Aesthetic check
		4	Check of the primary telescope, fixation impression
		5	Complete check
		6	Insertion of the definitive denture • Cementation of the primary telescope (Ketac™ Cem, 3M ESPE, Neuss, Germany)
Removable denture	Jaw bar	1	Open impression
		2	Abutment check
		3	Jaw relation (wax splint)
		4	Aesthetic check
		5	Jaw bar check
		6	Complete check
		7	Finishing
	Ball attachment	1	Impression of the edentulous alveolar ridge
		2	Jaw relation (wax splint)
		3	Aesthetic check
		4	Chairside insertion of the matrices

All treatments were provided by two experienced maxillofacial surgeons (WR, CH) and two experienced prosthodontists (RS, JH) to minimise bias.

### Follow-up investigation

The first clinical follow-up was arranged at the latest 4 weeks after prosthetic treatment was completed. Further follow-ups were scheduled quarterly in the first year and later every 6 months. Patients were screened clinically and radiologically (yearly) for biological and technical complications. The authors applied the abovementioned success criteria according to Buser et al. [32]. Crestal bone changes were evaluated on digital radiograms (SIDEXIS imaging software, Sirona, Bensheim, Germany). The distance between the implant shoulder and first bone-implant contact at the mesial and distal aspect of each implant was measured (implant length as reference) by the first author (WR), and the mean values per implant were calculated [37] 1 and 2 years after loading.

### Data gathering and statistics

All patients were pseudonymised, parameters were attached to a databank and analysed statistically (Additional file 2). Statistical analyses were performed using statistics software (IBM SPSS statistics, version 20, Chicago, IL, USA). The descriptive statistics presented the frequency and distribution of several occurrences as well as combinations of certain features. Analytical statistics were performed depending on the scale: paired and independent *t* tests for differences of mean values. The implant survival was calculated according to the Kaplan-Meyer method. The level of significance was set at 5%.

## Results

The first results of this longitudinal study include data from 9 patients with an average age of 57 years (range from 44 to 80) in whom 30 implants were inserted (maxilla *n* = 15, mandible *n* = 15). All 30 implants in the 9 patients could be inserted without intraoperative problems. Based on intraoperative and radiological findings, the bone quality was assessed as follows: D1 in *n* = 2, D2 in *n* = 3, D3 in *n* = 2 and D4 in *n* = 2 cases. The employed implant dimensions were as follows: 4.1 × 5 mm (*n* = 2), 4.1 × 6 mm (*n* = 1), 4.1 × 7 mm (*n* = 10) and 3.75 × 7 mm (*n* = 17). The expansion process could successfully be performed in every case. The healing period was uneventful. Patients were rehabilitated with fixed dentures in 5 cases and with removable dentures in 4 cases. Basic clinical characteristics are summarised in detail in Table 4.

**Table 4:** Clinical characteristics of the study cohort

Patient	Sex	Age (years)	Implant position (*FDI*)	Indication category^a^	Bone quality	Prosthetic treatment	Follow-up (months)	Implant failure
1. T. I.	F	80	Maxilla 15, 13, 11, 21, 25 (Σ = 5)	IIa	D4	Telescope	37	*n* = 1^c^
2. G. S.	F	65	Mandible 34, 32, 42, 44 (Σ = 4)	IIIb	D1	Ball attachment	34	None
3. S. Sa.	F	64	Maxilla 14, 12, 22, 24 (Σ = 4)	IIIa^b^	D4	Jaw bar	34	None
4. Th. F.	M	76	Mandible 35, 36, 37 (Σ = 3)	IIb	D1	Bridge	33	None
5. A. M.	F	44	Maxilla 16, 15, 14 (Σ = 3)	IIa	D3	Bridge	32	None
6. S. M.	M	53	Maxilla 16, 14, 12 (Σ = 3)	IIa	D3	Ball attachment	32	*n* = 1^d^
7. K. S.	F	52	Mandible 35, 36, 37 (Σ = 3)	IIb	D2	Bridge	29	None
8. R. C.	F	59	Mandible 35, 36 (Σ = 2)	IIb	D2	Bridge	24	None
9. W. K.	F	72	Mandible 47, 45, 43 (Σ = 3)	IIb	D2	Bridge	23	None

*FDI* implant position according to the World Dental Federation

^a^Indication categories (IIa, IIb, IIIa, IIIb) with regard to the amount of implants [35]

^b^Modified due to local conditions

^c^Implant loss before loading

^d^Implant loss after loading

Over the 3-year follow-up period, the overall cumulative implant success rate in these patients was 28/30 (93.3%). Two implants were lost in the posterior maxilla. The two affected patients had highly atrophic posterior maxillae (Cawood et Howell IV–V) [38] and a bone quality of D3–D4 (Table 4). The male patient was a smoker and suffered from a squamous cell carcinoma of mouth floor. In both cases, the manufactured removable denture was successfully relined and no technical complications were observed to date.

**Fig. 2:**
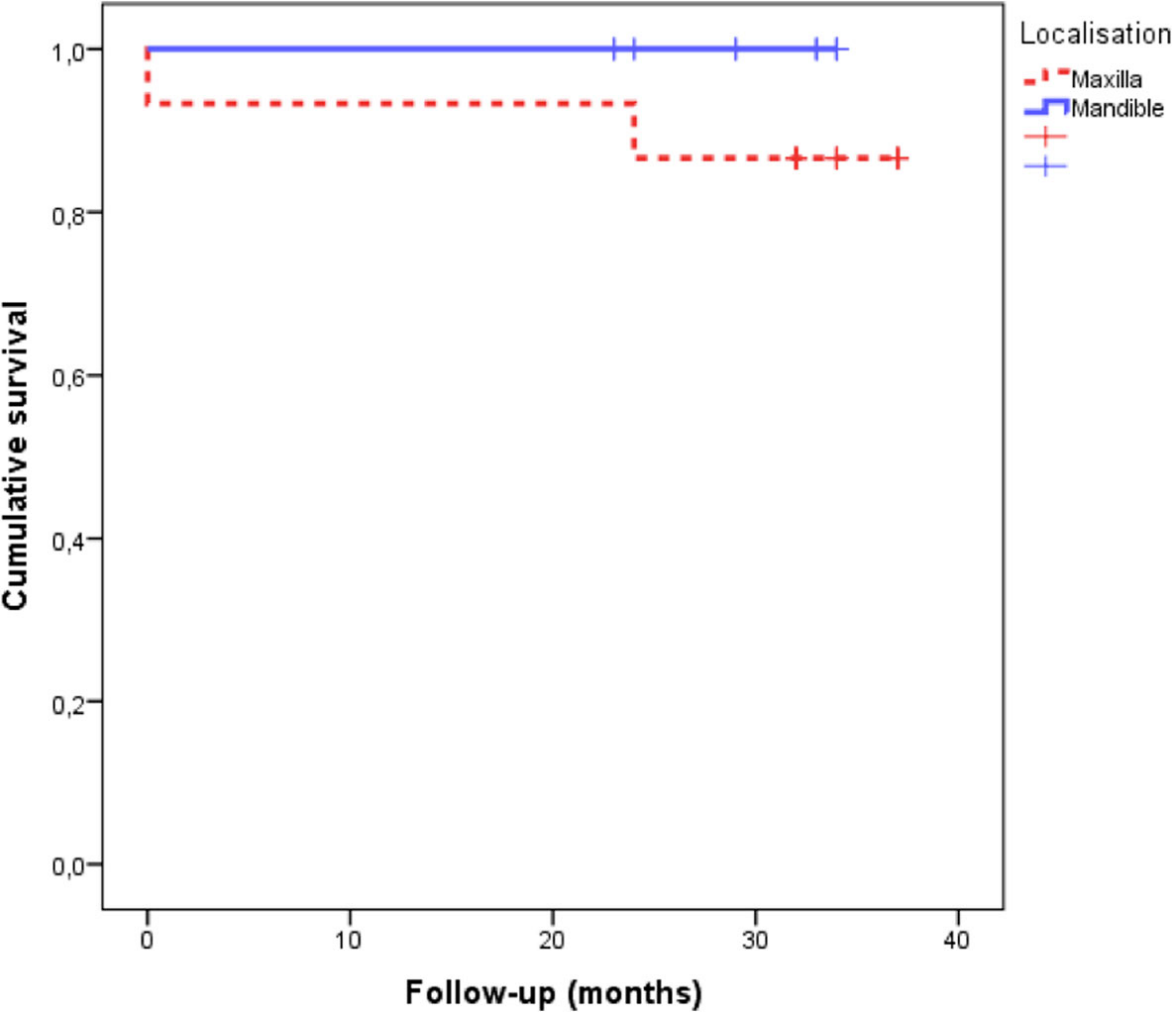
Cumulative implant survival over the follow-up period. The Kaplan-Meyer diagram visualises the analysis of implant survival in the maxilla and in the mandible (log rank test, *p* = 0.173) over the follow-up period up to 37 months (Table 4)

The Kaplan-Meyer analysis of implant survival for both jaws is visualised in Fig. 2 (log rank test, *p* = 0.173): 1-year survival 96.7% and 2-year survival 93.3%. The 3-year follow-up has not yet been completed by all patients (Table 4).

**Fig. 3:**
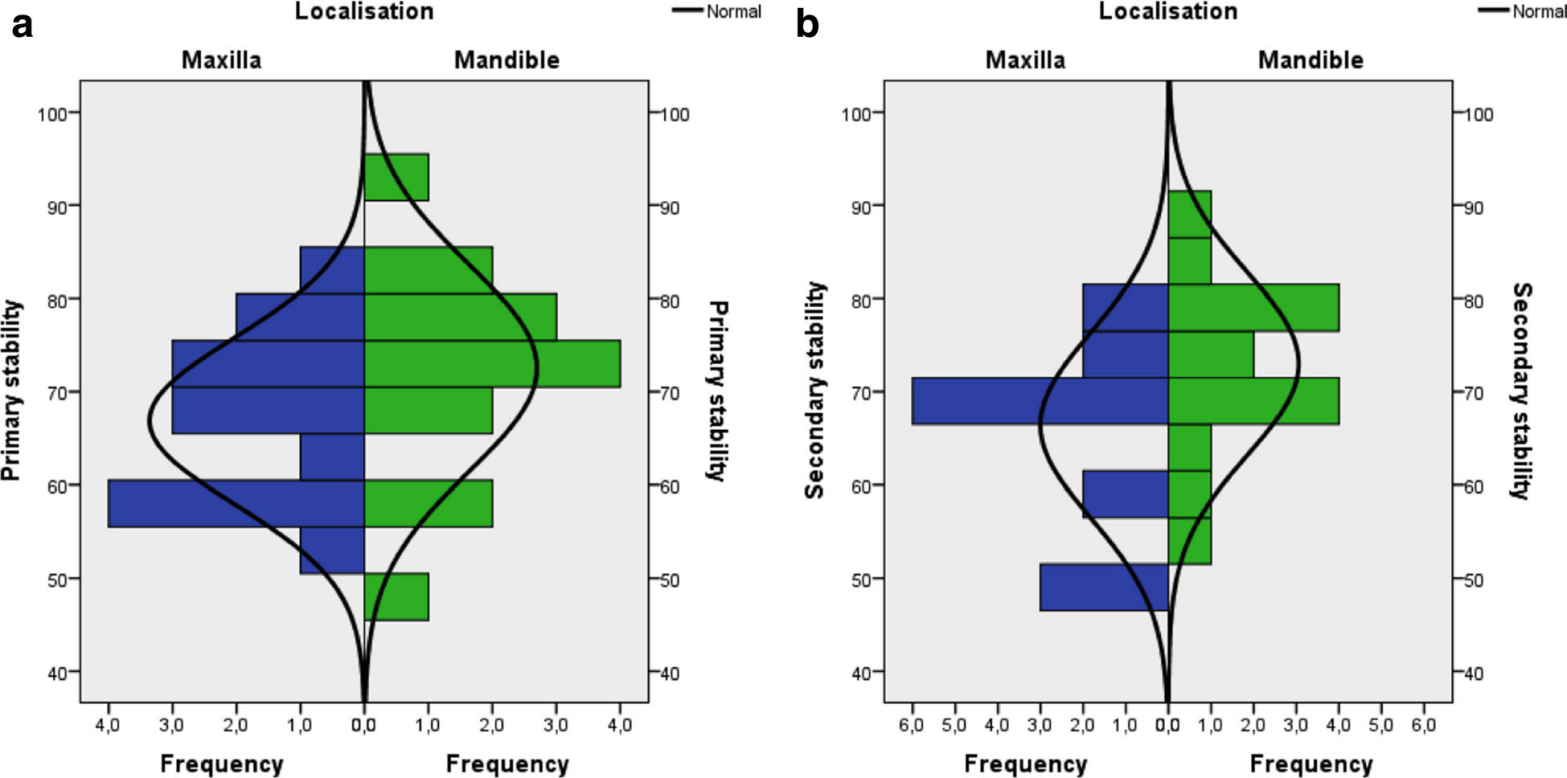
**a** Primary implant stability. The histogram visualises the distribution of the implant stability quotients (ISQ) for both jaws measured by resonance frequency analysis (Osstell AB, Göteborg, Sweden). **b** Secondary implant stability. The histogram shows the distribution of the implant stability quotients (ISQ) of osseointegrated implants. According to the measurement implant stability was classified as *low* with ISQ values < 60, *medium* with ISQ values 60–70, and *high* with values ISQ > 70 [34]

Measurements of implant stability by resonance frequency analysis (RFA) displayed the following ISQ values: primary stability 69.7 ± 10.3 95% CI (65.9; 73.6) ISQ units and secondary stability 69.8 ± 10.2 95% CI (65.8; 73.5) ISQ units (Fig. 3a, b). The differences were not statistically significant (*p* = 0.780; paired *t* test). In detail, the ISQ values for primary stability displayed in the maxilla 66.9 ± 8.9 95% CI (61.9; 71.8), and in the mandible 72.5 ± 11.1 95% CI (66.4; 78.7). The differences were not statistically significant (*p* = 0.134; independent *t* test). According to the measurement of secondary implant stability, we observed comparable ISQ values in the maxilla 66.4 ± 10.0 95% CI (60.9; 71.9) and higher ISQ values in the mandible 73.0 ± 9.7 95% CI (67.6; 78.4). The differences were as well not statistically significant (*p* = 0.780; independent *t* test).

Over the follow-up period, the mean crestal bone changes after loading were as follows (each compared to the baseline): in the first year, 1.0 ± 0.9 mm 95% CI (0.5; 1.5) in the maxilla and 0.7 ± 0.4 mm 95% CI (0.5; 1.0) in the mandible (*p* = 0.011; independent *t* test), and in the second year, 1.3 ± 0.8 mm 95% CI (0.8; 1.7) in the maxilla and 1.0 ± 0.7 mm 95% CI (0.6; 1.4) in the mandible (*p* = 0.644; independent *t* test). Clinical and radiological investigations did not reveal any inflammatory signs or radiolucency in the periapical region for all inserted implants.

**Fig. 4:**
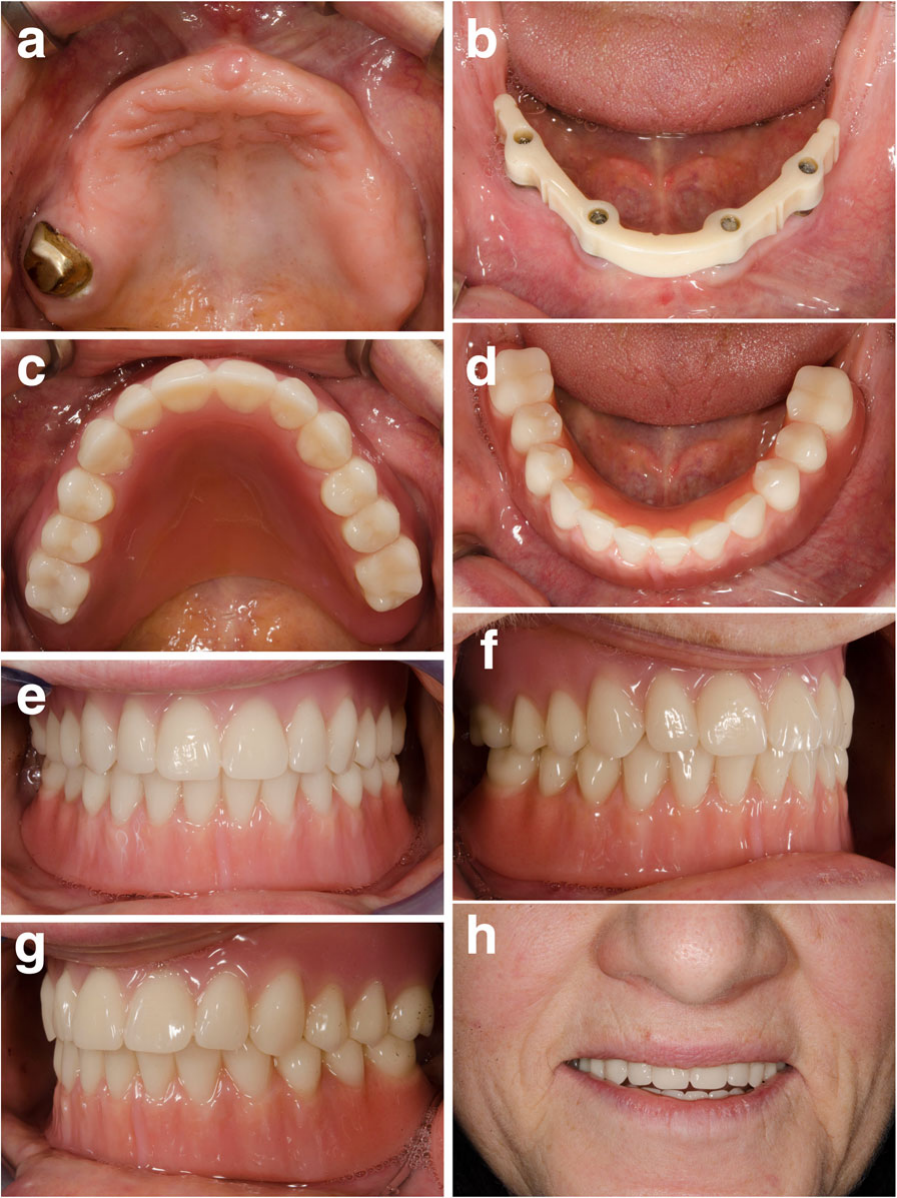
**a–h** Prosthetic restauration—follow-up examination. Intraoral and perioral views of a rehabilitated female patient. (She asked explicitly only for implantological treatment in the mandible.)

**Fig. 5:**
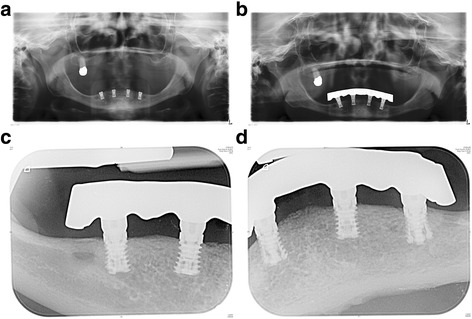
**a** Postoperative orthopantomogram. **b** Follow-up orthopantomogram. **c** Follow-up standard periapical radiogram (implants i42 and i44). **d** Follow-up standard periapical radiogram (implants i32 and i34)

A representative case of a rehabilitated female patient is visualised in Fig. 4a–h and Fig. 5a–d (radiograms).

## Discussion

Recent literature has shown that short implants have achieved growing acceptance in the field of oral implantology [9, 10, 39, 40]. Since the last years, concern has decreased about the length of endosseous implants; it should be noted that all extraoral screw implants are short implants [41, 42]. Nevertheless, there are local physiological and biomechanical differences regarding long-term stability.

The survival rate of short dental implants was found to increase from 80 to > 90% over time [39]. This is also confirmed in recent studies. For short dental implants supporting single crowns and fixed bridges especially in the mandible, a 2-year success rate of 97% [43] and a 5-year outcome of 92.2% [10] are reported. Otherwise, the success rate of 100% in the maxilla (3-year outcome) [15] must be critically questioned in view of our findings.

Only a few reports in the literature have addressed *expandable dental implants* [44–47]. In 2001, Jo and co-authors reported about a 40-month prospective survival of an expandable standard-length implant (10–16 mm) for immediate loading. They found a 3-year survival rate of 96.1% in the maxilla and of 94.8% in the mandible [46]. Huré and co-authors [47] performed a biomechanical and histologic canine study on early loaded expandable implants of 10- and 11.5-mm lengths. Six years later (2010), in orthopaedic surgery, an expandable implant was introduced [48]. Similar with the present study, these authors addressed implants under difficult regional bony conditions.

The purpose of our study was to evaluate the intraoperative handling, safety and feasibility of a new *expandable dental implant* system in a heterogeneous study cohort. We found in the present pilot study an overall 3-year implant success rate of 93.3%, which is comparable with recent literature [39]. To the best of our knowledge (PubMed), the present clinical study is the first published investigation about the usage of an *expandable short dental implant system*. Therefore, directly comparable data from other clinical studies are missing.

Several investigators analysed the preferred indications of short dental implants in the posterior mandible or maxilla and outlined the cost efficiency compared to additional vertical augmentations. In the present trial, we used a new short implant in both jaws and nearly all possible indication categories were represented, which proves the broad versatility (Table 4).

In our study, two implant failures occurred early in the prosthetic period and under loading. In a former systematic review, 11 studies reported more short implant failures *before* loading, while 7 studies reported more implant failures *after* loading [39]. Regarding the implant success rate in the present study, it must be considered that the lost implants were associated with difficult surgical conditions. Besides biological failures, in this study cohort, no technical complications were observed. In accordance with earlier comparative studies, it is evident that when using short implants, there is a lower risk of complications compared to augmentation [4, 7, 8] and nerve lateralisation [40].

Why design modifications? This is a matter of reduction of the healing period [46], the gain of stability under difficult conditions and increased bone-to-implant contact [27, 49–51] and the fact that most complications of short implants occur in the *preprosthetic period* [39]. It is also a question of long-term crestal bone stability. Earlier biomechanical finite-element studies confirmed that apical expansion results in favourable stress reduction in the *crestal bone* of nearly 10% [52]. It is assumable that additionally to the microthread and platform-switching concept [53], the periimplant bone strain could be reduced by apical expansion. This issue requires separate consideration in further studies. The employed implant design (especially its 7-mm length) combines several favourable biomechanical features, which were considered in this study (Fig. 1a, d).

According to Gehrke and co-authors, and in relation to the present study, the *apical implant design* influences the implant stability and bone-to-implant contact [54]. The expansion procedure presents an additional bicortical anchorage [17] in the oro-vestibular direction. In hard bone, this might be a disadvantage and lead to asymmetrical expansion. Manufacturers’ recommendations for hard bone should be strongly considered.

Regarding resonance frequency analysis, the values are related to bone quality and quantity as well as the exposed implant height above the alveolar crest, which depends on the type of implant and insertion technique [55–57]. Our results (primary stability in the maxilla 66.9 ± 8.9 ISQ units and in the mandible 72.5 ± 11.1 ISQ units; secondary stability in the maxilla 66.4 ± 10.0 ISQ units and in the mandible 73.0 ± 9.7 ISQ units) are comparable with the results from Becker and co-authors (standard-length implants): primary stability 72.1 ISQ units and secondary stability 72.6 ISQ units [58]. These values are marginally lower than those of short implants inserted only in the *posterior mandible* (79.0 ISQ units) [12]. Other authors measured in the *posterior maxilla* 68.2 ISQ units (6-mm implants) [15]. Altogether, our mean results (Fig. 2a, b) represent high stability values [34]. Huré and co-authors [47] measured in their animal study the following stability values (expandable implant of ≥ 10-mm length): for primary stability, 53.6 ± 3.0 ISQ units, and for secondary stability (3 months after insertion), 59 ± 4.5 ISQ units. The evaluation of stability values during the osseointegration period was not possible in our trial due to submerged healing. The question, whether the level of implant stability achieved at insertion can be maintained during the early healing period, remains. This should be analysed separately for all bone types in front of the known lowest stability values at 3–4 weeks after placement for all bone types [59–61] and the recent attempts of immediate [14] or early (6 weeks) functional loading of other short implant systems [28]. In relation to the results by McCullough and Klokkevold [62], who found that the macrothread design appears to play a positive role in implant stability in the early healing period, this can also be assumed for the employed implant system. Additionally, with regard to the results by Marković and co-authors [61], a critical stability drop down due to bone remodeling after bone condensing (implant site preparation and/or using expandable implants) should not be suspected; the opposite can be expected. The authors analysed the implant stability (4.1 × 10-mm screw implant) in the posterior maxilla in vivo depending on the implant site preparation (bone condensing vs. bone drilling) and confirmed that, after bone condensing, significantly higher implant stability results were achieved, immediately after implant insertion as well as during the whole observation period of 6 weeks. Especially in the third week in both groups, the following results were measured: 66.7 ± 1.64 vs. 57.1 ± 1.45 (*p* < 0.001). [61]. In the present study, we measured in the posterior maxilla 66.3 ± 10.4 ISQ units for primary stability and 66.9 ± 12.0 ISQ units for secondary stability, respectively.

Contrary to conventional hollow-screw implants (only marginal gap), a problem of the expandable implant is the presence of gaps down to the apical region. Former microbial assessment of different implant-abutment interfaces displayed that none of the marginal connections had the capacity to prevent microbial leakage [63–65]. Therefore, an apical microleakage (comparable to distractable implants and endodontically treated teeth) might be a disadvantage of the evolved implant system [66, 67]. However, according to the manufacturer’s information, a microbiological study revealed no microbial leakage through the expanded implants. Over the follow-up period, we equally did not observe any inflammatory signs in the apical region, neither clinically nor radiologically (Figs. 4b and 5c–d). Nevertheless, this aspect should be analysed under mechanical loading in vitro. Based on an earlier animal histologic study [47], as well as a clinical up-to-40-month study [46], which referred to comparable apically expandable implants, authors did not report any periapical inflammatory complications. To eliminate the potential risk of deep intrabony microleakage, it is questionable whether equal biomechanical stability values can be achieved only by the macrothread design avoiding any deep microgaps.

In the present study, the crestal bone changes under loading in the first year exceeded that of the second year. Moreover, the differences between the maxilla and mandible in each year were not statistically significant, which only partially agrees with previous findings in the literature [7, 58]. Besides microbiological conditions, there are several biomechanical aspects which influence maintenance of periimplant crestal bone. Conical and parallel surfaces of the implant-abutment connection (internal hexagon) provide rotational stability and reduce microgaps and micromovement [68]. Another important factor is the thickness of the implant shoulder [69], which might be a weak point in the design of a short implant due to elastic deformity under *extra-axial* loading. This fact might be the reason for non-inflammatory periimplant crestal bone loss. We addressed this aspect by splitting adjacent implants wherever possible [50, 51]. According to Brenner and co-authors [30] as well as Pommer and co-authors [50], the following *prosthodontic factors* are to be considered to avoid screw loosening, component fracture, loss of marginal bone or even loss of osseointegration: crown-to-implant ratio (extra-axial loading), cantilever length, status of opposing dentition, splinting of adjacent implants, occlusal surface relief and dimensions.

Comparable studies displayed at 24 months a crestal bone loss of 0.5–0.6 mm [15]. Other authors reported at 2, 3 and 5 years a mean loss of 0.57, 0.55 and 0.53 mm, respectively, in the mandible (without significant change after 1 year) [10]. On the other hand, randomised controlled trials demonstrated 1 year after loading periimplant marginal bone loss of 0.7 mm [70] and 1.1 mm [7] in the mandible which is the same value measured in the present study.

Within the limitations of a pilot study design, low number of implants, single-arm study and short-term follow-up, the results show a basic improvement of functional rehabilitation especially for elderly patients with compromised general and local conditions for implantation. Controversial questions [5, 71] remain on whether (a) short implants are suitable for irradiated patients and (b) there is a need for expandable short implants in the D1 bone. Furthermore, potential bias should be eliminated in future studies by a randomised controlled trial.

## Conclusion

Initial results of the ongoing study confirm the feasibility and safety of the employed system. The implant type seems to be useful for all bone qualities and shows high initial and secondary biomechanical stability in the maxilla and mandible. Long-term follow-up will be needed in validating these initial results in a larger 3-year clinical trial. Crestal bone changes should be evaluated in a larger study cohort. The novel system might extend the spectrum in functional rehabilitation.

## Additional files

Additional file 2: Dataset presenting relevant raw data. (SAV 3 kb)

## Abbreviations

D1, D2, D3, D4: Bone quality (density)
FDI: World Dental Federation
ISQ: Implant stability quotient
RFA: Resonance frequency analysis
